# Intra-abdominal sepsis in critically ill surgical patients: the relationship between cumulative fluid balance and serum sodium and chloride levels and in-hospital mortality

**DOI:** 10.3389/fmed.2025.1608388

**Published:** 2025-07-16

**Authors:** Radmila Popović, Nada Anđelić, Gordana Jovanović, Sanja Maričić Prijić, Arsen Uvelin, Nataša Tomić, Aleksandra Plećaš Ðurić, Nemanja Todorović, Boris Milijašević, Dejan Marković

**Affiliations:** ^1^Department of Anesthesiology and Perioperative Medicine, Faculty of Medicine, University of Novi Sad, Novi Sad, Serbia; ^2^Clinic for Anesthesia, Intensive Care and Pain Management, University Clinical Center of Vojvodina, Novi Sad, Serbia; ^3^Department of Nursing, Faculty of Medicine, University of Novi Sad, Novi Sad, Serbia; ^4^Department of Pharmacy, Faculty of Medicine, University of Novi Sad, Novi Sad, Serbia; ^5^Department of Pharmacology, Toxicology and Clinical Pharmacology, Faculty of Medicine, University of Novi Sad, Novi Sad, Serbia; ^6^Faculty of Medicine, University of Belgrade, Belgrade, Serbia; ^7^Center for Anesthesiology and Reanimatology, University Clinical Centre of Serbia, Belgrade, Serbia

**Keywords:** intra-abdominal sepsis, in-hospital mortality, fluid therapy, fluid balance, chlorides, sodium

## Abstract

**Background and aim:**

Intra-abdominal sepsis in critically ill surgical patients has a high mortality rate. Fluid therapy is essential resuscitation measure but can lead to poor outcome due to fluid overload and increased sodium and chloride levels. This study aimed to examine the relationship between cumulative fluid balance, serum sodium and chloride levels in the intensive care unit (ICU), and in-hospital mortality in critically ill surgical patients with intra-abdominal sepsis.

**Methods:**

The study was designed as a retrospective, observational study. Data were collected and analyzed from 100 critically ill surgical patients with intra-abdominal sepsis who were immediately subjected to surgical treatment. Postoperative care continued in the ICU for at least 7 days. Data related to daily fluid enteral and parenteral intake and loss were taken from medical records. The cumulative fluid balance was calculated for the periods from days 1 to 3 and 1 to 7 of ICU treatment.

**Results:**

In-hospital mortality rate was 51%. The cumulative fluid balance on the third and seventh days of ICU hospitalization was found to be positively correlated with mortality. Statistical analyses revealed significant differences in fluid balance at these time points in relation to mortality (*p* < 0.0005). ROC analysis confirmed the predictive power of cumulative fluid balance, with an AUC of 0.757 (cutoff: 5,130 ml, sensitivity 68.6%, specificity 69.4%) on the third day and AUC of 0.856 (cutoff: 2,210 ml, sensitivity 78.4%, specificity 83.7%) on the seventh day. Binary logistic regression further supported the influence of fluid balance on mortality. Sodium and chloride levels remained within the reference range but were significantly higher in patients who died. Binary logistic regression showed that abnormal sodium and chloride levels on the third and seventh days were associated with increased mortality.

**Conclusion:**

High values of postoperative cumulative fluid balance as well as elevated serum sodium and chloride levels during the first 7 days in the ICU may be important predictors of in-hospital mortality in critically ill patients with intra-abdominal sepsis who underwent emergency surgical treatment.

**Clinical trial registration:**

https://clinicaltrials.gov/study/NCT06838585?locStr=Novi%20Sad,%20Serbia&country$=$Serbia&state$=$Vojvodina&city$=$Novi%20Sad&cond$=$intra%20abdominal%20sepsis&rank$=$3, NCT06838585.

## 1 Introduction

Sepsis is one of the main public health issues due to its high incidence, mortality rate, long-term health consequences, and economic burden (1, 2). According to data from 2020, there were 11 million sepsis-related deaths worldwide, accounting for 20% of all global deaths (3). Sepsis mortality varies significantly (15%−56%) and depends on numerous factors: age, comorbidities, septic shock, and the availability of adequate medical care (4, 5). In developed regions, the 30-day mortality from sepsis is 24.4%, the 90-day mortality is 32.3%, and within the first 5 years after hospitalization, it can reach up to 74% (5, 6). In 2017, the World Health Assembly and the World Health Organization (WHA/WHO) adopted a resolution prioritizing the global reduction of the sepsis burden (2).

According to the data available at the time of manuscript preparation, sepsis was identified in 29.5% of critically ill patients hospitalized in the intensive care unit (ICU), with 18% of these patients having sepsis upon ICU admission. ICU and intrahospital mortality rates were 25.8 and 35.3%, respectively, although these rates varied by region (7). Certain subgroups of critically ill patients are more prone to sepsis. This cohort includes critically ill surgical patients, who exhibit an altered inflammatory response, different sources of sepsis, and specific risks associated with the perioperative period (8).

In surgical ICUs, intra-abdominal infections (IAI) are among the most common causes of sepsis (9, 10). The epidemiology of IAI and sepsis in critically ill patients was demonstrated in a multicenter, observational study by Blot et al. They found that, in a cohort of critically ill patients with IAI, 31.6% had community-acquired IAI, while the remaining patients had in-hospital IAI (25% early-onset hospital-acquired infection and 43.4% late-onset hospital-acquired). Overall mortality was 29.1%. Late-onset hospital-acquired infection, diffuse peritonitis, septic shock, and antimicrobial resistance of the causative agent were identified as independent mortality risk factors (11). Factors contributing to the risk of mortality in surgical patients include delayed diagnosis and treatment, the severity of infection (septic shock), comorbidities (such as diabetes mellitus, cardiovascular disease, and immunocompromised), inadequate and delayed control of the source of sepsis, antimicrobial resistance of pathogens, advanced age (>60 years), and the quality of treatment in the hospital and ICU (12, 13).

The basic therapeutic principles of treating patients with sepsis and septic shock include early recognition, adequate antibiotic therapy, source control, support of organ function, and early and intensive fluid resuscitation (14). For hemodynamic optimization, patients often receive significant amounts of fluid, leading to fluid overload and accumulation of sodium and chloride as complications (15). The most common complications associated with fluid overload include cardiovascular and respiratory insufficiency, pleural effusions, acute kidney injury, gastrointestinal failure, tissue edema, poor wound healing, wound infection, and high intra-abdominal pressure (16). A particularly vulnerable population of critically ill patients includes those with underlying state of fluid retention, such as chronic renal failure, acute kidney injury, and chronic heart failure (17). One of the first large studies to demonstrate a relationship between cumulative fluid balance and mortality in critically ill patients was the Sepsis Occurrence in Acutely Ill Patients (SOAP) study. The study found that cumulative fluid balance correlated with 60-day mortality and was one of the strongest prognostic factors for mortality (18).

Numerous studies have showed the serious harmful consequences of excessive intravenous fluid use in surgical patients (19, 20), but few have addressed this issue in the context of emergency surgery. One study indicated that a lower fluid balance (< 2.0 L) was associated with a reduced risk of cardiopulmonary complications in patients undergoing emergency surgery for gastrointestinal obstruction and perforation (21). Fluid overload in the postoperative period leads to lung congestion, respiratory insufficiency, reduced tissue oxygenation, impaired wound healing, edema, and prolonged recovery. Silva et al. (22) showed that fluid balance was an independent risk factor for mortality in patients treated in the ICU after major surgical procedures.

In patients with intra-abdominal sepsis who require urgent surgery, aggressive fluid resuscitation can lead to fluid accumulation in the abdomen, bowel edema, and increased intra-abdominal pressure (IAP). This will exacerbate the inflammatory response and raise the risk of complications (23). Therefore, it is essential to define the objectives of perioperative fluid therapy and use fluids as any other medication that requires a specific dose and timing for administration (24, 25).

Although sepsis has been investigated in numerous studies, there is still limited understanding of how fluid overload, sodium, and chloride levels contribute to mortality in patients with intra-abdominal sepsis, particularly those in the ICU. This study aims to examine their effect on in-hospital mortality in patients with intra-abdominal sepsis.

## 2 Materials and methods

### 2.1 Study population and ethics approval

A retrospective observational cohort study was conducted at the University Clinical Center of Vojvodina, specifically in the Surgical ICU at the Clinic for Anesthesia, Intensive Care, and Pain Management. The study included all adult patients who were admitted to the hospital and underwent emergency surgical treatment for community-acquired intra-abdominal sepsis. Postoperative care was continued in the surgical ICU for at least 7 days. The study period covered January 2020 to September 2024. Patients who were discharged from the ICU in < 7 days (either transferred to the hospital ward or who died during this period) were excluded from the study. Furthermore, patients presenting with intra-abdominal sepsis and an additional concurrent infection upon admission, those previously hospitalized or operated on for other conditions within the preceding 30 days, pregnant women, and those with incomplete medical documentation were also excluded from the study. Only the first ICU admissions were considered for analysis.

The study was approved by the Ethics Board of the University Clinical Center of Vojvodina (approval number 00-108). In accordance with the ethical standards and Serbian legislation, informed consent from patients was not required given that the study was retrospective in nature and involved the analysis of data from medical records.

### 2.2 Data source

The data required for this study were obtained by reviewing medical documentation and the clinical information system. Demographic data (age and gender), preoperative Sequential Organ Failure Assessment (SOFA) and Acute Physiology and Chronic Health Evaluation II (APACHE II) scores were recorded. Furthermore, the presence of septic shock on admission and during the first 7 days, as well as acute kidney injury on admission and during the first 7 days of ICU hospitalization, were also assessed. The number of days from the onset of symptoms to hospitalization was noted, along with laboratory values for serum sodium and chloride preoperatively and on the third and seventh days of hospitalization. Data regarding the length of hospitalization in the ICU and total length of hospital stay were collected, as well as the number of days of mechanical ventilation. Complications recorded included reoperations (surgical complications requiring reoperation) and intrahospital infections. Given the aim of the study, intrahospital mortality was monitored.

### 2.3 Definitions

The diagnosis of sepsis and septic shock is based on the Third International Consensus Definitions for Sepsis and Septic Shock (Sepsis-3) (26).

Acute kidney injury (AKI) was diagnosed when the patient met the criteria for AKI stage 1 or higher within the first week of ICU admission (27).

### 2.4 Daily fluid status and cumulative fluid balance

The data on the daily balance of fluid intake and loss were extracted from temperature charts, where these data are recorded daily. Total daily fluid intake included parenteral fluids (crystalloids, colloids, blood and blood derivatives, parenteral nutrition, fluids for dissolving medicines, and those used for maintaining intravenous lines), as well as enteral and peroral intake during the first 7 days of ICU treatment. Intraoperative intravenous fluid administration was also considered. Total daily fluid losses were categorized into diuresis, losses through drains and nasogastric tubes, ultrafiltrate after RRT, and insensible fluid losses. Cumulative fluid balance was calculated for the following periods: from day 1 to day 3, from day 4 to day 7, and for the entire first week (days 1–7) of ICU treatment. Intraoperative fluid balance was also included for the first day of hospitalization. The daily fluid balance for each patient was determined by subtracting the total fluid losses over a 24-h period from the total fluid intake over the same period. Cumulative fluid balance for the first week was calculated for the following periods: days 1–3, days 4–7, and days 1–7.

### 2.5 Aim of the study

This study aimed to examine whether there was an association between cumulative fluid balance and serum sodium and chloride levels on the third and seventh days of hospitalization in the intensive care unit and in-hospital mortality in critically ill surgical patients with intra-abdominal sepsis who underwent emergency surgical treatment.

### 2.6 Statistical analysis

Statistical analysis was performed using SPSS Statistics 23.0 software. Data are presented as arithmetic mean, standard deviation, lowest value (minimum), highest value (maximum), number and percentage (%) depending on the type of data. The normality of the distribution of continuous variables was assessed using the Kolmogorov–Smirnov test. Depending on the type and normality of the distribution of the variables, the comparison of the differences between the investigated groups was carried out using appropriate parametric and non-parametric tests. To compare the mean values of variables of two populations, the *t*-test for independent samples and the Mann–Whitney test were used. The association of categorical variables was examined using the Chi-square test for contingency tables or Fisher's test. The Pearson correlation coefficient was used to examine the strength of the connection between the variables. Determining the impact of variables on the outcome of treatment was done using binary logistic regression. |In addition to the primary predictor variable (cumulative fluid balance), the following covariates were included due to their clinical relevance and potential confounding effects: patient age, sex, SOFA and APACHE II scores, presence of septic shock and acute kidney injury, time from symptom onset to hospitalization, serum sodium and chloride, ICU and total hospital stay length, duration of mechanical ventilation, reoperations, and intrahospital infections. These variables were selected based on previous evidence indicating their association with ICU outcomes in critically ill septic patients. The predictive quality of variables on the outcome was assessed using ROC curves. The results are presented tabular and graphically, and values are considered statistically significant at the *p* < 0.05 level.

## 3 Results

A total of 100 patients (53 males and 47 females) who were admitted to the ICU at the University Clinical Center of Vojvodina following surgical intervention for severe intra-abdominal infection complicated by sepsis were included in this study. The patient characteristics in relation to survival outcomes (mortality) are presented in Table 1. Compared to survivors, deceased patients were significantly older (70.59 ± 10.58 vs. 63.29 ± 15.08), exhibited higher SOFA [5.0 (3.0–7.0) vs. 9.0 (7.50–10.0)] and APACHE II scores [14.50 (11.0–19.50) vs. 22.0 (19.0–26.0)]. The deceased had a substantially higher incidence of septic shock upon admission (94.1 vs. 38.8%), AKI (92.2 vs. 53.1%), and required longer durations of mechanical ventilation (*p* < 0.0005). Surviving patients experienced fewer complications during treatment, particularly surgical complications (35.3 vs. 0%), as well as intra-hospital infections (*p* < 0.0005). Additionally, deceased patients had a longer ICU stay, while survivors had a longer total length of treatment.

**Table 1:** Patient characteristics in relation to survival (mortality).

**Patient characteristics**	**Total sample (*N* = 100)**	**Survivors (*N* = 49)**	**Deceased (*N* = 51)**	***p*-value**
**Basic characteristics**				
Male	53 (53%)	26 (53.1%)	27 (52.9%)	0.990
Age	67.01 ± 13.42	63.29 ± 15.08	70.59 ± 10.58	0.006
SOFA score^*^	7.0 (5.0–10.0)	5.0 (3.0–7.0)	9.0 (7.50–10.0)	< 0.0005
APACHE II score^**^	19.0 (14.0–24.0)	14.50 (11.0–19.50)	22.0 (19.0–26.0)	< 0.0005
Number of days since the onset of symptoms	4.0 (3.0–5.0)	4.0 (2.0–4.0)	4.0 (3.0–5.0)	0.154
Septic shock upon admission	67 (67%)	19 (38.8%)	48 (94.1%)	< 0.0005
AKI	73 (73%)	26 (53.1%)	47 (92.2%)	< 0.0005
**Complications**				
Surgical complications	18 (18%)	0 (0%)	18 (35.3%)	< 0.0005
Intra-hospital infections	45 (45%)	10 (20.4%)	35 (68.6%)	< 0.0005
**Treatment outcome**				
ICU stay (days)^***^	9.0 (8.0–12.0)	9.0 (7.50–10.0)	10.0 (9.0–14.50)	< 0.0005
Hospital stay (days)	14.0 (11.25–17.0)	15.0 (13.0–17.0)	12.0 (9.0–16.50)	0.001
Ventilator day (days)	5.0 (3.0–10.0)	3.0 (2.0–3.0)	9.0 (8.0–13.0)	< 0.0005
In-hospital mortality	51 (51%)	NA	51 (51%)	

^*^SOFA score, sequential organ failure assessment score; ^**^APACHE II score, acute physiology and chronic health evaluation II score; ^***^ICU, intensive care unit.

An overview of the fluid balance during the first week of ICU stay, compared by survival outcomes, is presented in Table 2. The fluid balance was positive during the first 2 days of ICU stay in both groups. From the third to the seventh day, the fluid balance became negative in the survivors, while in the deceased group, it was negative only on the fourth day and positive on the remaining days. During the first 3 days of ICU stay, both groups exhibited a positive cumulative fluid balance, though it was significantly lower in the survivors (*p* < 0.0005). From the fourth to the seventh day, the cumulative fluid balance was negative in the survivors and positive in the deceased group (*p* < 0.0005). Overall, Table 2 demonstrates that the survivors had a significantly lower fluid balance compared to the deceased ones.

**Table 2:** Overview of fluid balance and cumulative fluid balance during the first week of ICU stay, compared based on survival outcomes.

**Fluid balance**	**Total sample (*****N*** = **100)**			**Survivors (*****N*** = **49)**			**Deceased (*****N*** = **51)**			***p*-value**
	**Input (ml)**	**Output (ml)**	**Input/output balance (ml)**	**Input (ml)**	**Output (ml)**	**Input/output balance (ml)**	**Input (ml)**	**Output (ml)**	**Input/output balance (ml)**	
Day 1	5,565 (4,400–6,542.5)	1,825 (1,330–2,860)	3,560 (2,212.5–4,895)	5,300 (4,085–6,125)	1,550 (1,265–2,740)	3,150 (1,740–4,640)	5,845 (4,750–7,395)	2,120 (1,560–2,900)	3,900 (2,880–5,060)	0.022
Day 2	4,085 (3,400–4,785)	2,975 (2,187.5–3,917.5)	1,197.5 (188.75–2,080)	3,620 (3,150–4,325)	3,370 (2,670–4,355)	620 (−240 to 1,655)	4,300 (3,850–5,180)	2,770 (1,980–3,800)	1,870 (1,055–2,470)	< 0.0005
Day 3	3,265 (2,885–3,800)	3,495 (2,515.5–4,185)	−120 (−987.5 to 945)	3,100 (2,610–3,755)	3,730 (3,050–4,160)	−450 (−1,110 to 195)	3,600 (2,130–4,290)	3,200 (2,130–4,290)	530 (−550 to 1,560)	0.002
Day 4	2,925 (2,240–3,595)	3,310 (2,720–4,267.5)	−460 (−1,376.25 to 155)	2,550 (2,077.5–3,150)	3,430 (3,032.5–4,150)	−800 (−1,490 to −365)	3,380 (2,700–3,925)	3,030 (2,300–4,330)	−120 (−800 to 1,040)	< 0.0005
Day 5	2,695 (2,046.25–3,400)	3,405 (2,692.5–3,995)	−565 (−1,555 to 447.5)	2,410 (1,995–2,880)	3,540 (3,135–4,025)	−1,060 (−1,675 to −380)	3,030 (2,100–3,880)	2,950 (1,940–3,940)	300 (−1,140 to 1,145)	< 0.0005
Day 6	2,825 (2,122.5–3,465)	3,185 (2,660–3,975)	−560 (−1,542.5 to 246.25)	2,450 (2,050–3,075)	3,600 (309–4,280)	−1,150 (−1,900 to −560)	3,100 (2,490–3,770)	2,660 (1,750–3,400)	200 (−700 to 780)	< 0.0005
Day 7	2,800 (2,057.5–3,330)	3,330 (2,422.5–3,972.5)	−660 (−1,287.5 to 415)	2,500 (1,985–3,200)	3,680 (3,175–4,305)	−1,030 (−1,710 to −720)	3,000 (2,100–3,690)	2,430 (1,360–3,500)	400 (−350 to 1,100)	< 0.0005
**Cumulative fluid balance**										
Day 1–3	12,895 (11,348.75–153,600)	8,395 (6,915–9,915)	5,055 (2,315–7,007.5)	11,970 (10,545–13,865)	9,330 (7,375–10,150)	2,820 (1,220–5,507.5)	13,730 (11,950–16,874)	7,525 (6,290–9,190)	6,160 (4,330–8,080)	< 0.0005
Day 4–7	10,987.5 (9,365–14,022.5)	13,325 (9,952.5–16,536.25)	−2,277.5 (−5,562.5 to 1,841.25)	10,170 (8,552.5–11,865)	14,500 (13,000–16,747.5)	−4,880 (−6,125 to −2,990)	12,700 (10,270–14,750)	10,020 (8,210–16,300)	1,635 (−1,750 to 3,440)	< 0.0005
Day 1–7	23,937.5 (21,329.25–28,227.5)	22,177.5 (17,118.75–26,250)	2,870 (−2,952.5 to 7,811.25)	22,530 (20,245–24,096)	23,090 (21,110–26,542.5)	−1,970 (−4,750 to 2,620)	26,870 (23,400–29,860)	18,590 (14,470–24,660)	7,360 (3,170–10,480)	< 0.0005

There is a moderate positive correlation between the cumulative fluid balance (days 1–3) and the mortality outcome (*r* = 0.398; *p* < 0.0005). Similarly, a moderate positive correlation is observed between the cumulative fluid balance (days 1–7) and the mortality outcome (*r* = 0.599; *p* < 0.0005).

The average cumulative fluid balance for days 1–3 in survivors was 3,035 (780–5,460) ml, while in deceased patients it was 6,160 (4,342.50–7,875) ml (*p* < 0.0005). For days 1–7, the fluid balance was −2,105 (−4,940 to 550) ml in survivors and 6,267 (2,835–9,992.50) ml in deceased patients (*p* < 0.0005). Additionally, there was a statistically significant difference in the mean values of Na and Cl in relation to in-hospital mortality. On days 3 and 7, Na levels were 142.00 (140.00–144.00) and 141.00 (139.00–145.00) in survivors, and 145.00 (141.00–148.00) and 148.00 (143.50–155.00) in deceased patients, respectively (*p* = 0.002 and *p* < 0.0005). On days 3 and 7, Cl levels were 109.00 (107.00–111.00) and 109.00 (103.00–110.00) in survivors, and 111.00 (108.00–115.00) and 113.00 (109.50–119.00) in deceased patients, respectively (*p* = 0.007 and *p* < 0.0005), as shown in Table 3.

**Table 3:** Differences in the mean values of cumulative fluid balance (days 1–3 and 1–7) and the mean values of Na and Cl (days 3 and 7) in relation to overall in-hospital mortality.

**Fluid balance parameter**	**Mortality outcome**	**Percentiles**			***p*-value**
		**25th**	**50 (median)**	**75th**	
Cumulative fluid balance, days 1–3	No	780.00	3,035.00	5,460.00	< 0.0005
	Yes	4,342.50	6,160.00	7,875.00	
Cumulative fluid balance, days 1–7	No	−4,940.00	−2,105.00	550.00	< 0.0005
	Yes	2,835.00	6,267.00	9,992.50	
Na, day 3	No	140.00	142.00	144.00	0.002
	Yes	141.00	145.00	148.00	
Na, day 7	No	139.00	141.00	145.00	< 0.0005
	Yes	143.50	148.00	155.00	
Cl, day 3	No	107.00	109.00	111.00	0.007
	Yes	108.00	111.00	115.00	
Cl, day 7	No	103.00	109.00	110.00	< 0.0005
	Yes	109.50	113.00	119.00	

**Figure 1:**
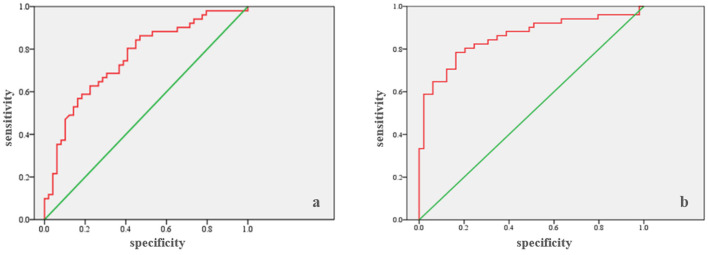
Sensitivity and specificity: **(a)** days 1–3, **(b)** days 1–7.

ROC analysis (Table 4) showed that the cumulative fluid balance for days 1–3 can be a marker for predicting in-hospital mortality (Area = 0.757; *p* < 0.0005), with a cut-off of 5,130 ml. The sensitivity is 68.6%, and the specificity is 69.4% (Figure 1a). Similarly, ROC analysis showed that the cumulative fluid balance for days 1–7 is a good marker for predicting in-hospital mortality (area = 0.856; *p* < 0.0005), with a cut-off of 2,210 ml. The sensitivity is 78.4%, and the specificity is 83.7% (Figure 1b).

**Table 4:** ROC analysis for cumulative fluid balance (days 1–3 and 1–7).

**Area under the curve**				
**Area**	**Std. error**	***p*** **-value**	**Asymptotic 95% confidence interval**	
			**Lower bound**	**Upper bound**
**Test result variable(s): cumulative fluid balance, days 1–3**				
0.757	0.048	< 0.0005	0.663	0.852
**Test result variable(s): cumulative fluid balance, days 1–7**				
0.856	0.039	< 0.0005	0.779	0.779

There is a moderate positive correlation between Na levels on both day 3 (*r* = 0.338; *p* = 0.001) and day 7 (*r* = 0.485; *p* < 0.005) and mortality. A weak positive correlation exists between Cl levels on day 3 (*r* = 0.265; *p* = 0.008) and in-hospital mortality, while a moderate positive correlation is observed between Cl levels on day 7 (*r* = 0.432; *p* < 0.0005) and in-hospital mortality.

Na levels on day 3 are associated with in-hospital mortality (*p* = 0.034). The percentage of deceased patients with Na levels within the reference range on day 3 is 43.3%, while 66.7% of those with Na levels outside the reference range died. Similarly, Na levels on day 7 are associated with mortality (*p* < 0.0005), with 34.5% of deceased patients having Na levels within the reference range and 71.1% having Na levels outside the reference range. Cl levels on day 3 (*p* = 0.009) and on day 7 (*p* < 0.0005) are both associated with mortality. The percentage of deceased patients with Cl levels within the reference range on day 3 is 42%, compared to 71% in those with Cl levels outside the reference range. The percentage of deceased patients with Cl levels within the reference range on day 7 is 34.8%, compared to 82.4% in those with Cl levels outside the reference range (Table 5).

**Table 5:** Comparison of Na and Cl levels on days 3 and 7, with reference ranges, and their association with total in-hospital mortality.

**Variable**	**Mortality outcome**		***p*-value**
	**No**	**Yes**	
**Na, day 3**			0.034
Within the reference range	38 (56.7%)	29 (43.3%)	
Outside the reference range	11 (33.3%)	22 (66.7%)	
**Na, day 7**			< 0.0005
Within the reference range	36 (65.5%)	19 (34.5%)	
Outside the reference range	13 (28.9%)	32 (71.1%)	
**Cl, day 3**			0.009
Within the reference range	40 (58%)	29 (42%)	
Outside the reference range	9 (29%)	22 (71%)	
**Cl, day 7**			< 0.0005
Within the reference range	43 (65.2%)	23 (34.8%)	
Outside the reference range	6 (17.6%)	28 (82.4%)	

Binary logistic regression analysis demonstrated that sodium levels on day 3 significantly influenced in-hospital mortality (*p* = 0.030), with an odds ratio of 2.621 (95% CI: 1.098–6.257). Patients with sodium levels outside the reference range on day 3 had a 2.6-fold higher risk of mortality compared to those with sodium levels within the reference range. Similarly, sodium levels on day 7 were significantly associated with mortality (*p* < 0.0005), with an odds ratio of 4.664 (95% CI: 1.991–10.924). Patients with sodium levels outside the reference range on day 7 had a 4.7-fold increased risk of mortality compared to those within the reference range. Chloride levels on day 3 also had a significant impact on mortality (*p* = 0.009), with an odds ratio of 3.372 (95% CI: 1.356–8.385). Patients with chloride levels outside the reference range on day 3 had an almost 3.5-fold higher risk of mortality compared to those within the reference range. Furthermore, chloride levels on day 7 were a strong predictor of mortality (*p* < 0.0005), with an odds ratio of 8.725 (95% CI: 3.156–24.117). Patients with chloride levels outside the reference range on day 7 had an 8.7-fold increased risk of in-hospital mortality compared to those with chloride levels within the reference range.

## 4 Discussion

The administration of intravenous fluids in critically ill patients is a crucial therapeutic measure. However, fluid and electrolyte overload, particularly of sodium and chloride, is a serious complication of this therapy, negatively impacting treatment outcomes. It also serves as an indicator of the severity of the underlying condition (28). Our study found that cumulative fluid balance and pathological values of sodium (Na) and chloride (Cl), monitored during the early days of hospitalization in the ICU, influence the mortality of surgical critically ill patients with intra-abdominal sepsis.

The results indicate a high in-hospital mortality rate of 51% among our patients, with an average length of hospitalization of 14 days (range: 11.25–17 days). The deceased patients were treated for a statistically significantly longer duration in the ICU (*p* < 0.0005), while the survivors had a longer overall treatment duration, as presented in Table 1. However, other studies have reported lower in-hospital mortality rates of 32.5% (29), 25.4% (30), and 24.1% (31). A meta-analysis by Bauer et al. on mortality in septic shock and sepsis over a 10-year period found that the average 30-day mortality for septic shock was 34.7%, with 90-day mortality at 38.5%. The average 30-day mortality for sepsis was 24.4%, and the 90-day mortality was 32.2% (5). Another study indicated that the mortality rate of patients with severe intra-abdominal infection and sepsis was ~30%. This high mortality was attributed to the significant proportion of patients with sepsis and organ dysfunction (32). Among the known risk factors for mortality in intra-abdominal sepsis, our patients exhibited the following: delayed operative care due to late presentation to the healthcare facility, cumulative fluid balance, sodium and chloride overload, impaired renal function (present in 73% of patients), and septic shock (affecting more than 60% of patients) (11). Considering the aforementioned factors, we particularly emphasize that patients presented for examination, after which immediate urgent operative treatment and ICU admission were carried out, on average 4 (3–5) days after the onset of symptoms. This delay is notably long, and according to current understanding of the causes of high mortality in intra-abdominal sepsis, it could be a significant contributing factor to the mortality observed in our patients (33, 34).

ROC analysis showed that the cumulative fluid balance for the 1–3 day period can be a marker for predicting mortality, with a cut-off value of 5,130 ml (sensitivity 68.6%, specificity 69.4%). The same conclusion can be drawn for the 1–7 day period, with a cut-off value of >2,210 ml (sensitivity 78.4%, specificity 83.7%). Binary logistic regression showed that patients with the specified cut-off values for cumulative fluid balance on day 3 have an almost five times higher risk of mortality compared to patients with a lower cumulative fluid balance. For the 1–7 day period, the risk of mortality is 14.5 times higher.

In a retrospective study by Sim et al. a cut-off value of 20 ml/kg/day was established. Patients with a postoperative fluid load ≥20 ml/kg/day had a higher risk of developing respiratory complications and increased 30-day mortality. However, since the study was retrospective, a causal relationship between positive fluid balance, fluid load, organ dysfunction, and mortality could not be established. Further statistical analysis indicated that the positive fluid balance was not attributed to the severity of the disease or oliguria, but rather to the administration of a larger volume of intravenous fluids. Similar to our patient population (septic, surgical critically ill patients), this study highlights the complexity of fluid management–balancing an adequate amount of fluid to optimize the patient's condition without overloading them with unnecessary fluid (30). The 2021 global Surviving Sepsis Campaign guidelines recommend an initial fluid bolus of 30 ml/kg for resuscitating patients with septic shock (14). However, the optimal fluid amount for initial resuscitation in emergency surgical care remains debated. Some studies support “early liberation, late conservation” fluid strategy, which is associated with the lowest mortality. Patients with a lower daily fluid balance have better outcomes, particularly in terms of 30-day mortality. Prolonged liberal fluid therapy during the perioperative period can negatively impact critically ill surgical patients (30).

In a prospective study by Acheampong and Vincent, which included 173 patients (a mix of non-surgical and surgical patients) treated for sepsis in the ICU, the relationship between positive fluid balance and its maintenance during ICU hospitalization was analyzed as an independent prognostic factor. Total fluid intake in the first 3 days averaged 11.8 L (157 ml/kg), lower than in our study (5,130 ml). Daily fluid intake was higher in patients who died compared to survivors (*p* = 0.03), with no difference in daily fluid losses (*p* = 0.49). Overall, daily fluid balance was higher in those who died (*p* < 0.01) (35), similar to our data. A positive fluid balance was independently associated with increased ICU mortality risk. Our findings indicate that a positive cumulative fluid balance observed on days 3 and 7 was correlated with in-hospital mortality. Furthermore, the study by Acheampong and Vincent showed that initially, fluid balance was similar in both survivors and those who died, but from day 2 onward, it became more positive in the deceased. The difference in fluid balance was due to higher fluid administration in patients with poor outcomes, rather than lower losses. The study also examined the use of diuretics and renal replacement therapy, finding that the association between positive fluid balance and mortality persisted regardless of these interventions (35). In our study, this correlation was not addressed.

A systematic review and meta-analysis conducted by Messmer et al. evaluated 31 observational studies and three randomized controlled trials to assess the impact of fluid loading, defined as a weight gain exceeding 5% or a positive cumulative fluid balance, on mortality in critically ill adult patients. The overall conclusion of the study was that fluid loading is associated with increased mortality in critically ill patients, as well as in specific subpopulations, including those with sepsis, acute renal failure, respiratory failure, and postoperative conditions. The authors also concluded that for each additional liter of cumulative fluid balance, the adjusted risk of mortality increased by a factor of 1.19 (95% CI, 1.11–1.28) (28). In our study, a quantitative effect was also observed, as the average cumulative fluid balance for both measurement periods demonstrated a statistically significant difference between survivors and non-survivors.

A retrospective study conducted by Lee et al. investigated the association between fluid balance at ICU discharge and mortality in a cohort of nearly 16,000 subjects (both nonsurgical and surgical patients) treated in the ICU of a large, tertiary medical center. The conclusion of this study was that a positive fluid balance during ICU hospitalization is associated with a significantly higher risk of death within 90 days of ICU discharge. This association was particularly pronounced in patients with chronic heart failure, acute renal failure, and impaired renal function–conditions that predispose individuals to fluid retention. Furthermore, the relationship between positive fluid balance and increased mortality was independent of the maximum fluid balance values during ICU hospitalization. In subjects with the highest maximum fluid balance values, a return to normal pre-discharge fluid balance at discharge was associated with improved survival. The study by Li et al. emphasized the importance of careful monitoring of fluid balance during ICU hospitalization, particularly at the time of discharge. The magnitude of fluid balance is a modifiable factor that can be managed by the treating physician (17).

Initial fluid administration in patients with septic shock aims to optimize hemodynamics and typically involves the administration of larger volumes (30 ml/kg). However, due to insufficient evidence, there are currently no definitive recommendations on whether to adopt a restrictive or liberal fluid strategy in patients who, despite initial resuscitation, fail to achieve adequate perfusion (14). The CLASSIC study by Meyhoff is one of the most recent large, international, randomized trials to investigate various strategies for intravenous therapy in patients with septic shock. The median value of administered intravenous fluids in the restrictive group was 1,798 ml (IQR: 500–4,366 ml), while in the standard group it was 3,811 ml (IQR: 1,861–6,762 ml). Ninety-day mortality in the restrictive group was 42.3%, while in the standard group it was 42.1%. In comparison to the median cumulative fluid balance on the fifth day in our study, which had a negative value of −565 ml, the CLASSIC study reported values of 1,676 ml in the restrictive group and 2,420 ml in the liberal group (36). The differences in these values can be attributed to several factors: in our study, no distinction was made between patients with sepsis and septic shock, whereas the CLASSIC study specifically focused on patients with septic shock. Additionally, the latter study did not specify whether it included surgical or non-surgical critically ill patients.

A prospective, multicenter, observational study by Wang et al. identified a trend in fluid balance among septic patients and found that this trend was associated with in-hospital mortality and the development of organ dysfunction during the first 7 days after ICU admission. In this study, 20.1% of patients were fluid-loaded, and their risk of in-hospital death was 1.4 times higher compared to patients without fluid overload. Fluid accumulation was observed to be gradual in the group with low fluid balance, while it occurred more rapidly in the group with high fluid balance. Fluid balance during the first 24 h of ICU hospitalization was not associated with intrahospital mortality, which supports the conclusions of previous studies suggesting that early fluid resuscitation is linked to a reduced risk of in-hospital mortality in patients with sepsis and septic shock (31, 37).

The complexity of the patient population in our study arises from the fact that, in addition to being septic, these patients were also surgical. Therefore, their management must be considered within the context of the perioperative period. We will review several studies that have addressed perioperative fluid administration and its impact on postoperative outcomes.

In the observational study by Sribar et al. the cohort consisted of surgical patients with endocarditis who were treated postoperatively in the ICU. The median cumulative fluid balance during ICU hospitalization in the study was 1,190 ml, which is comparable to our data (1,532 ml for the first 7 days of hospitalization). Among the surviving patients, 56.4% had a cumulative fluid balance lower than the median, while 70% of those who died had a cumulative fluid balance higher than the median. If the SOFA score and patient age were considered, patients with a cumulative fluid balance greater than the median demonstrated nearly a threefold higher risk of in-hospital mortality. The mortality rate in the studied population was 15% (38). In our study, ROC analysis indicated that cumulative fluid balance can serve as a marker for predicting mortality (area = 0.757; *p* < 0.0005), with a cut-off value of 5,130 ml (sensitivity 68.6%, specificity 69.4%) for the first 3 days of ICU hospitalization. The risk of death was five times higher when the cumulative fluid balance exceeded 5,130 ml. For the first 7 days, the risk of death was 14.5 times higher if the cumulative fluid balance exceeded 2,210 ml. In the study conducted by Sribar et al. a statistically significant correlation was found between the duration of mechanical ventilation and ICU length of stay, although no such correlation was established between these parameters and cumulative fluid balance. This study highlighted the impact of cumulative fluid balance during ICU hospitalization on in-hospital mortality in a surgical population of patients with endocarditis (38).

Wu et al. conducted a retrospective cohort study at a tertiary medical center in Taiwan, spanning from 2015 to 2019. In surgically critically ill patients admitted to the ICU, in-hospital mortality was 10.3%, 90-day mortality was 23.8%, and 1-year mortality was 31.7%. Compared to survivors, a higher positive cumulative fluid balance was observed in the first 3 days (1,607.0 ± 3,326.5 ml vs. 920.8 ± 2,266.1 ml, *p* < 0.01) in those who died. A similar trend was observed for the 4–7day period (269.5 ± 2,300.3 ml vs. −145.4 ± 1,523.2 ml, *p* < 0.01). Very similar findings were obtained in our study. The conclusion of the study by Wu et al. was that fluid balance during the first week of hospitalization in critically ill surgical patients, particularly during the 4–7day period, can influence the long-term outcomes of their treatment (29).

A large retrospective cohort study by Van Regenmortel and colleagues aimed to identify and quantify all sources and indications for fluid administration in critically ill patients. The study included over 14,500 critically ill patients, with surgical patients comprising ~52%. Chloride loading was specifically addressed in the study, as chloride levels can be reduced by using hypotonic solutions to maintain volume. In septic patients, fluids administered for purposes other than resuscitation were found to have a significant impact on the cumulative fluid balance. The movement of the average cumulative fluid balance values was as follows: on day 1, 520 ± 1,531 ml; on day 3, 2,223 ± 3,310 ml; and on day 5, 3,404 ± 4,613 ml (39). The values reported by Van Regenmortel et al. were highlighted because their patient population was the most similar to ours. Our study recorded the median values of fluid balance as follows: 3,560 ml on day 1, 5,130 ml on day 3, and 1,532 ml on day 7, which differ from those reported in their study. The nearly double value of the mean fluid balance on day 1 could be attributed to the inclusion of intraoperative fluid balance. For the other values, the differences may be explained by the fact that their study also included septic patients. The conclusion of the study emphasized the need to avoid unintentional daily intake of volume, sodium, and chloride when prescribing infusion therapy, due to the significant amounts of fluid creep (39).

In our study, the mean values of sodium (Na) and chloride (Cl) dominantly remained within reference limits (Na: 135–145 mmol/L; Cl: 96–112 mmol/L) throughout all 7 days of hospitalization, though they showed an upward trend. The highest average values of these electrolytes were recorded on day 7 of hospitalization, with Na at 146.6 ± 7.11 mmol/L and Cl at 110.75 ± 6.78 mmol/L. A weak to moderate positive correlation was observed between the concentrations of these electrolytes measured on specific hospitalization days and the overall in-hospital mortality rate. Statistically significant differences in the mean values of Na and Cl were found on day 7 in relation to inhospital- mortality. Binary logistic regression analysis revealed that patients with sodium (Na) and chloride (Cl) values outside the reference range have a higher risk of death. In the following discussion, we will review several studies on sodium and chloride loading and examine the impact of these electrolyte imbalances on treatment outcomes and the occurrence of complications in critically ill patients.

A prospective observational study by Shirazy et al. demonstrated that hypernatremia on day 7 of ICU hospitalization was associated with increased mortality in patients with sepsis and septic shock. Hypernatremia observed on day 1 of hospitalization was associated with prolonged ICU stay (40). In the study by Shirazi et al. 18.7% of patients developed hypernatremia by day 7 of ICU hospitalization, while a study by Van De Louw et al. found that 31% of patients developed hypernatremia by day 5 of ICU admission (40, 41). The mean sodium (Na) concentrations in our study were slightly higher than in the study by Shirazy et al. but remained within the reference limits for the observed period.

A study by O‘Donoghue and colleagues explored acquired hypernatremia as an independent predictive factor for mortality in critically ill patients. The incidence of hypernatremia in a mixed population of critically ill patients was 7.7%. Intrahospital mortality in the hypernatremia group was 33.5%, compared to 7.7% in the group with normal sodium (Na) values (*p* < 0.001) (42). In our study, among the 51 patients who experienced in-hospital mortality, 66.7% had a sodium (Na) concentration disorder on day 3 of hospitalization, while 71.1% had this disorder by day 7. Of all patients with in-hospital mortality (51 patients), 66.7% had an abnormal sodium (Na) concentration on day 3 of hospitalization, while 71.1% had this abnormality on day 7 of ICU hospitalization. Acquired hypernatremia in the O'Donoghue study was an independent risk factor for intrahospital mortality. Intermediate Na values 145–150 mmol/L) were associated with increased mortality. The pathological value of Na at discharge from the ICU and the maximum value of Na were a better predictor of mortality than the time of onset and duration of the disorder (42).

In the study by Yeh et al. univariate statistical analysis revealed that increased peak serum chloride levels, hyperchloremia (≥110 mEq/L), a greater number of days with sustained hyperchloremia, and higher intravenous chloride intake were associated with increased ICU mortality, new acute kidney injury by day 7, and multiple organ failure by day 7 (43). Our study showed that a significant percentage of patients with a fatal outcome had elevated chloride (Cl) levels on both the days 3 and 7 of ICU hospitalization. The risk of in-hospital mortality was increased by 3.5 times on day 3 and 8.7 times on day 7 for pathological Cl values.

A retrospective observational study by Gwak et al. found that patients with a fatal outcome (22.2%) had significantly higher serum chloride levels compared to survivors (139.7 ± 8.1 vs. 119.1 ± 10.4 mmol/L; *p* < 0.001). Additionally, it was shown that every 5 mmol/L increase in peak serum chloride concentration elevated the risk of in-hospital mortality (aOR, 4.34; 95% CI, 1.98–9.50; *p* < 0.001) (44). Similar to this study, our research also demonstrated that patients with hypochloremia, observed on the days 3 and 7 of ICU hospitalization, had higher in-hospital mortality rate.

In the early phase of septic shock, the administration of a large volume of intravenous fluids is indicated in most patients to optimize organ perfusion. However, this often results in fluid overload as a complication. This is particularly pronounced in cases where adequate hemodynamic monitoring to assess the patient's responsiveness to infusion therapy has not been implemented (15, 45). However, it has been shown that only half of septic patients are fluid responders (46). Consequently, assessing which patients will benefit from fluid administration is crucial. It has been shown that optimizing fluid therapy based on static parameters (such as central venous pressure, pulmonary artery occlusion pressure, etc.) is unreliable (47). To adequately assess the response to fluid administration, dynamic parameters based on heart-lung interaction are used. These rely on measuring changes in stroke volume or cardiac output caused by variations in preload. The most commonly used dynamic tests are pulse pressure variation (PPV), stroke volume variation (SVV), end-expiratory occlusion test (EEOT), inferior vena cava respiratory variability, and passive leg raising test (PLR) (48, 49).

## 5 Conclusion

Elevated postoperative cumulative fluid balance, alongside increased serum sodium and chloride levels during the first 7 days in the intensive care unit, may serve as significant predictors of in-hospital mortality among critically ill patients with intra-abdominal sepsis who have undergone emergency surgical intervention.

## Data Availability

The raw data supporting the conclusions of this article will be made available by the authors, without undue reservation.
